# Sensory Impairment is Associated With Recurrent Falls: Study of Women’s Health Across the Nation

**DOI:** 10.1093/geroni/igab046.2901

**Published:** 2021-12-17

**Authors:** Carrie Karvonen-Gutierrez, Michelle Hood, Joshua Ehrlich, Richard Neitzel, Kelly Ylitalo

**Affiliations:** 1 University of Michigan, School of Public Health, Ann Arbor, Michigan, United States; 2 University of Michigan, Ann Arbor, Michigan, United States; 3 University of Michigan, University of Michigan, Michigan, United States; 4 University of Michigan School of Public Health, Ann Arbor, Michigan, United States; 5 Baylor University, Waco, Texas, United States

## Abstract

This study evaluated the relationship between individual and combined sensory impairments (vision, hearing, peripheral nerve (PN)) with recurrent falls in the past year among 1951 women (mean age 65.6 years) from the Study of Women’s Health Across the Nation. Sensory impairments were defined as self-reported vision difficulty, hearing loss, or ≥4 on the Michigan Neuropathy Screening Instrument. Recurrent falls were defined as ≥2 self-reported falls. Hearing was the most commonly reported impairment (39.2%), followed by vision (22.1%) and PN (16.0%). Among those with any impairments, 7.0% of women reported impairments in all domains. Recurrent falls were more common among women with vision (19.4%), hearing (17.3%), or PN impairments (24.7%) as compared to women without sensory impairments (7.0%). The greatest burden of recurrent falls was among women with all three sensory impairments; one-third (34.6%) of women with vision, hearing and PN impairment were recurrent fallers. In an adjusted logistic regression model, vision, hearing, and PN impairments were associated with statistically significantly higher odds of recurrent falls in the past year (odds ratio (OR) = 1.58, 1.76, 2.11, respectively; all p<0.01), after adjustment for age, race/ethnicity, economic strain, and depressive symptoms. The presence of all three sensory impairments was associated with nearly 6-fold increased odds of recurrent falls (OR=5.65, 95% CI 3.25, 9.82) compared to women with no impairments. Sensory impairments often onset during mid-life and early late adulthood. This work demonstrates that these impairments are associated with falls and that women with impairments across multiple sensory domains are at greatest risk.

